# Calcium-sensitive regulation of monoamine oxidase-A contributes to the production of peroxyradicals in hippocampal cultures: implications for Alzheimer disease-related pathology

**DOI:** 10.1186/1471-2202-8-73

**Published:** 2007-09-16

**Authors:** Xia Cao, Zelan Wei, Geraldine G Gabriel, XinMin Li, Darrell D Mousseau

**Affiliations:** 1 The Cell Signalling Laboratory, Neuropsychiatry Research Unit, University of Saskatchewan, 103 Wiggins Road, Saskatoon, SK S7N 5E4, Canada

## Abstract

**Background:**

Calcium (Ca^2+^) has recently been shown to selectively increase the activity of monoamine oxidase-A (MAO-A), a mitochondria-bound enzyme that generates peroxyradicals as a natural by-product of the deamination of neurotransmitters such as serotonin. It has also been suggested that increased intracellular free Ca^2+ ^levels as well as MAO-A may be contributing to the oxidative stress associated with Alzheimer disease (AD).

**Results:**

Incubation with Ca^2+ ^selectively increases MAO-A enzymatic activity in protein extracts from mouse hippocampal HT-22 cell cultures. Treatment of HT-22 cultures with the Ca^2+ ^ionophore A23187 also increases MAO-A activity, whereas overexpression of calbindin-D28K (CB-28K), a Ca^2+^-binding protein in brain that is greatly reduced in AD, decreases MAO-A activity. The effects of A23187 and CB-28K are both independent of any change in MAO-A protein or gene expression. The toxicity (*via *production of peroxyradicals and/or chromatin condensation) associated with either A23187 or the AD-related β-amyloid peptide, which also increases free intracellular Ca^2+^, is attenuated by MAO-A inhibition in HT-22 cells as well as in primary hippocampal cultures.

**Conclusion:**

These data suggest that increases in intracellular Ca^2+ ^availability could contribute to a MAO-A-mediated mechanism with a role in AD-related oxidative stress.

## Background

MAO-A and MAO-B, two isoforms of monoamine oxidase (MAO), are expressed on the mitochondrial outer membrane. MAO-mediated neurodegeneration can result from the formation of hydrogen peroxide (H_2_O_2_) as a by-product of metabolism of aminergic neurotransmitters including serotonin and dopamine. If it is not detoxified by antioxidant systems such as glutathione peroxidase – one of the most abundant such systems in brain [1] – then H_2_O_2 _can be converted by iron-mediated Fenton reactions to hydroxyl radicals that can initiate lipid peroxidation and cell death. This is exacerbated when antioxidant systems are compromised, such as during aging [2]. The reduction in the efficacy of these systems may simply be aggravated in chronic disease states such as Alzheimer disease (AD)[3].

It has been demonstrated that inhibitors of MAO-B, such as l-deprenyl and, more recently, rasagiline, are effective in the management of early symptoms of Parkinson's disease in the clinic and in animal models [4-6] as well as in patients with mild AD-type dementia [4,7]. Both MAO-B-positive astrocytes [8] and reactive oxygen species (ROS) [9] have been found in the vicinity of β-amyloid (Aβ) plaques. L-Deprenyl and rasagiline, however, may exert some of their effects independently of MAO-B as the neuroprotection mediated by these drugs is often associated with concentrations of the drug that are well below those required for inhibition of the enzyme [6], and has been associated with activation of Bcl-2 family members, interactions with the mitochondrial pore complex, and modulation of amyloid precursor protein cleavage [4].

MAO-A also plays a role in neuropsychiatric and behavioral disorders. The importance of this isoform is suggested by the aggressive phenotype seen in male mice deficient in MAO-A [10] and in males in a Dutch kindred bearing a spontaneous mutation (resulting in a premature stop) in the *mao-A *gene [11]. Similarly, maltreated children, whose genotype confers low levels of MAO-A expression, more often develop conduct disorder, antisocial personality and adult violent crime than do children with a high-activity MAO-A genotype [12]. Relatively modest changes in MAO-A activity/function can have important neuropsychiatric consequences as demonstrated by the fact that [^11^C]-harmine-labeled MAO-A is elevated by only 34% throughout the brain of untreated depressed patients compared to controls, yet it appears to be the major contributor to monoamine metabolism in these same patients [13]. Depression not only may promote cognitive impairment, but also may be a risk factor for AD [14]. Not surprisingly, MAO-A, which is often targeted for the treatment of depression, is also a potential risk factor for late-onset AD [15-18]. In contrast to irreversible inhibitors of MAO-A such as clorgyline, reversible inhibitors such as moclobemide are better tolerated and have been particularly efficacious in treating depression [19,20] and cognitive disorders [21] in the elderly. In addition, they protect against apoptosis [22] and glutamate-induced excitotoxicity [23]*in vitro*. Inhibition of MAO-A activity protects against striatal damage produced by the mitochondrial poison malonate and appears to rely on attenuation of dopamine-derived ROS [24], while apoptosis following serum starvation is reduced in MAO-A deficient cortical brain cells [25].

The NMDA-subtype of glutamate receptor has been linked to massive increases in mitochondrial Ca^2+ ^accumulation [26]. It is interesting that activation of this same receptor has been linked to Ca^2+^-dependent processes and pathology in [hepatic] encephalopathic brains [27] that correlates with a selective increase in MAO-A activity [28]. The selectivity of Ca^2+ ^for MAO-A is demonstrated *ex vivo *using brains from monkey [29], mouse [30] and rat [31], and is supported by the ability of the Ca^2+^-channel antagonist nimodipine to block the selective increase in MAO-A activity observed in senescence-accelerated mouse brain [32]. Additional cations able to influence MAO-A function include Zn^2+^, which inhibits MAO-A activity [29], and Al^3+ ^(another ion often linked to AD-like pathology), which activates it [33].

These combined observations, in addition to the fact that cytoplasmic free Ca^2+ ^is elevated in aged neurons and even more so during neurodegeneration, such as that encountered during in AD [15,34-36], certainly argue for examination of the relation between Ca^2+ ^and MAO.

## Methods

### Antibodies and reagents

5-Hydroxytryptamine (5-HT), β-phenylethylamine (PEA), the β-actin antibody and protease inhibitor cocktail were bought from Sigma-Aldrich Co. [^14^C]-5-HT (NEC-225) and [^14^C]-PEA (NEC-502) were purchased from PerkinElmer Life Sciences. The MAO-A and CB-28K antibodies were purchased from Santa Cruz Biotechnology. IgG-HRP conjugates were obtained from Cedarlane Laboratories. All other reagents were purchased commercially.

### Immortalized hippocampal cell cultures

The immortalized mouse hippocampal HT-22 cell line [20] was kindly provided by Dr. P. Maher (The Scripps Research Institute, La Jolla, CA, USA). Cells were cultured (5% CO_2 _at 37°C) in DMEM/low glucose medium containing 10% fetal bovine serum, 100 IU/mL penicillin G sodium salt and 0.03% glutamine.

### Monoamine oxidase (MAO) activity

MAOA and -B activities (nmol/hr/mg protein) in HT-22 cell cultures were estimated radiochemically [37]. Briefly, samples were homogenized in 80 volumes of oxygenated potassium phosphate buffer (0.2 M, pH 7.8) and incubated (100 μg/100 μl) for 10 min at 37°C with either 250 μM [^14^C]-5-HT (for MAO-A activity) or 50 μM [^14^C]-β-PEA (for MAO-B activity). The incubation was terminated by acidification and the labeled metabolites were extracted into ethyl acetate/toluene (1:1 vol/vol, water-saturated), an aliquot of which was subjected to scintillation spectrometry to determine radioactive content. MAO-A substrate kinetics was determined using protein (100 μg per reaction) from HT-22 cells pre-incubated in the absence or presence of Ca^2+ ^(1 mM; 20 min, room temperature) and subsequently incubated with increasing concentrations of [^14^C]-5-HT [15 μM-4 mM]. Incubation in oxygenated potassium phosphate buffer (0.2 M, pH 7.8) and extraction of labeled metabolites proceeded as described above. Data were analyzed for estimates of K_m _and V_max _using the Prism v3.01 software.

### Transient overexpression of CB-28K

The pREP-CB-28K (calbindin-D28K) plasmid expression vector was kindly  provided by Dr. A. Pollock (University of California, San Francisco, CA). HT-22 cells were seeded in log phase and transfected with plasmid DNA (1–2 μg/well on a 24-well plate; seeded at 5 × 10^5 ^cells/well) using ExGen™500 (Fermentas) according to the manufacturer's directions. Expression of eGFP fluorescent protein revealed a transfection efficiency of approximately 50% using this technique. Cells were routinely harvested 24 h post-transfection.

### Immunodetection of target proteins

Treated HT-22 cells were washed twice with ice-cold PBS and proteins were extracted in ice-cold lysis buffer (1% Triton X-100, 10% glycerol, 1 mM EDTA, 20 mM Tris, pH 7.5), containing protease inhibitor cocktail and 1 mM orthovanadate. Standard denaturing (SDS-PAGE) conditions were used to resolve proteins, which were then transferred to nitrocellulose. Protein expression in total cell lysates (20–30 μg/lane, precleared; 5000 × *g*, 10 min, 4°C) was visualized by enhanced chemiluminescence. Depicted immunoblots are representative of two-three independent experiments.

### Determination of target mRNA by semi-quantitative reverse transcriptase-PCR (RT-PCR)

Total RNA from treated HT-22 cells was prepared with TRIZOL reagent according to the manufacturer's protocol and digested with RNase-free DNase to clear residual genomic DNA. First strand cDNA was reverse-transcribed from 2 μg of total RNA using oligo-(dT) (SuperScript™III First-Strand Synthesis System, Invitrogen). The cDNA was amplified using *Taq *polymerase and primer pairs (*mao-A*: forward: 5'-GAA GCT GAG CTC TCC TGT TAC-3'; reverse: 5'-ACA AAG CAG AGA AGA GCC AC-3'; *mao-B*: forward: 5'-GCT GAA GAG TGG GAC TAC ATG AC-3'; reverse: 5'-GGA ATG AAC CTT GGG AGG TG-3'; β-*actin*: forward: 5'-TAG AAG CAT TTG CGG TGC ACG-3'; *reverse*: 5'-TGC CCA TCT ATG AGG GTT ACG-3'). The resultant semi-quantitative PCR products were electrophoresed on agarose gel and visualized using ethidium bromide staining.

### Fluorescence determination of free intracellular Ca^2+^

HT-22 cells were loaded with the fluorescent dye Fluo3-acetoxymethyl ester (Fluo-3-AM; Molecular Probes) at 4 μM for 30 min at 37°C [36]. Fluorescence was visualized at an excitation wavelength of 488 nm and an emission wavelength of 550 nm on an Olympus FV300 Confocal Laser Scanning Biological Microscope.

### Visualization of cytoplasmic peroxide radicals

2',7'-Dihydrodichlorofluorescein diacetate (DCFH2-DA) [37] permeates the cell membrane and is hydrolyzed to DCFH2, a nonfluorescent compound that remains trapped within the cell, but which yields a fluorescent product upon oxidation by H2O2. The cells were seeded on cover-slips. After treatment, the cells were rinsed twice with PBS and incubated with DCFH_2_-DA (5 μM, 30 min at 37°C) or DMSO, vehicle). The cells were washed in prewarmed HEPES-buffered (20 mM) HBSS (pH 7.0) containing 5 mM glucose, and prepared for DCF fluorescence (excitation: 488 nm; emission: 530 ± 15 nm).

### Rat primary hippocampal culture

Animal care followed protocols and guidelines approved by University of Saskatchewan Animal Care Committee and the Canadian Council on Animal Care. Rat hippocampal cultures were prepared from E18 fetuses (time-pregnant Sprague-Dawley rats; Charles River Canada, Montreal, PQ, Canada) as described before with some modifications [38]. In brief, the hippocampus was dissected in Ca^2+^- and Mg^2+^-free Hank's balanced salt solution (HBSS) supplemented with 15 mM HEPES and penicillin (100 U/ml)-streptomycin (100 μg/ml) (Gibco). Collected tissues were digested at 37°C with 0.25% trypsin-EDTA 15 min. The reaction was quenched with fetal bovine serum (FBS, 10%) and tissues were rinsed 3–4 times with HBSS to remove FBS. Following centrifugation at 800 × g for 10 min, the medium was removed and cells were resuspended in a chemically defined serum-free NeuroBasal medium supplemented with 1% N_2_, 2% B27, 50 μM L-glutamine, 15 mM HEPES, 10 U/ml penicillin and 10 μg/ml streptomycin. Neurons were then plated on coverslips (coated with 25 μg/ml poly-D-lysine), and grown at 37°C with 5% CO_2_-humidified atmosphere. The medium was replaced 24 h later with fresh NeuroBasal medium lacking L-glutamine and antibiotics. Medium was replaced after 4–5 days *in vitro *(DIV). Neurons were treated on DIV 7, following which they were fixed with 4% paraformaldehyde in 0.01 M PBS for 20 min at room temperature, washed several times with PBS, and stained with Hoechst 33258 (500 ng/ml, 10 min) for microscopic visualization of chromatin condensation (a characteristic of apoptotic cell death).

### Statistical analyses

Significance (set at *P *< 0.05) was assessed by unpaired *t*-tests or by one-way ANOVA with *post hoc *analyses relying on Bonferonni's Multiple Comparison Test (GraphPad Prism v3.01). Data are represented as mean ± standard deviation (SD).

## Results

### Low millimolar concentrations of Ca^2+ ^increase MAOA activity in HT-22 cells

**Figure 1:**
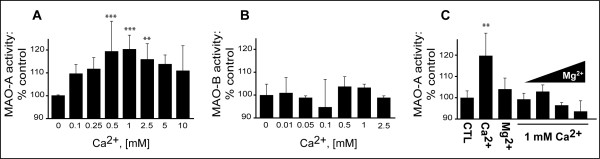
**Effect of Ca^2+ ^on MAO activity in the mouse hippocampal HT-22 cell line**. HT-22 cell lysates (100 μg/100 μL; n = 4) were incubated with increasing concentrations of Ca^2+ ^and assayed radioenzymatically for (A) MAO-A and (B) MAO-B activities. (C) HT-22 lysates (n = 3) were incubated with either Ca^2+ ^or the Ca^2+ ^antagonist Mg^2+ ^(1 mM) or co-incubated with Ca^2+ ^(1 mM) and increasing concentrations of Mg^2+ ^to test for the potential of Mg^2+ ^to block Ca^2+ ^(1 mM)-sensitive MAO-A activity. **: *P *< 0.01, ***: *P *< 0.001 vs. control. Data represent mean ± SD.

MAO-A activity in HT-22 cell extracts incubated with Ca^2+ ^(range: 0.1–10 mM) was increased, with a peak (~20% above baseline) around 0.5–1 mM Ca^2+ ^(Fig. 1A). Ca^2+ ^did not exert any effect on MAO-B activity (Fig. 1B). Mg^2+ ^on its own did not exert any effect on MAO-A activity, but it did inhibit the effect of Ca^2+ ^(Fig. 1C).

**Figure 2:**
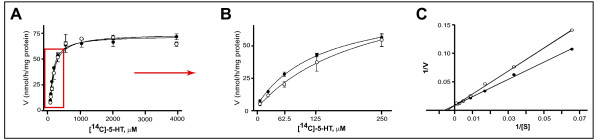
**Effect of Ca^2+ ^on MAO-A substrate kinetics in HT-22 hippocampal cell line**. (A) HT-22 cell lysates (100 μg/100 μL; n = 3) were incubated with increasing concentrations of substrate, *e.g*. [^14^C]-5-HT in the absence (○) or presence (●) of Ca^2+ ^(1 mM). The individual curves represent data pooled from three independent experiments. Data are presented as mean ± SD. (B) The highlighted part of the curves in (A) is expanded to demonstrate the change in K_m_. (C) Data presented on a double-reciprocal plot clearly demonstrate a change in slope (*e.g*. K_m_/V_max_) with no change in *y*-intercept (*e.g*. 1/V_max_).

Examination of the kinetics of MAO-A activity in the presence of Ca^2+ ^revealed a decrease in K_m _(μM) [97.6 ± 6.99 with Ca^2+ ^*vs*. 125.8 ± 21.02 without Ca^2+^; n = 3, *P *= 0.0462, *t *= 2.203, df = 4], indicating that Ca^2+ ^facilitates the enzymatic reaction. V_max _(nmol/h/mg protein) remained unchanged [74.4 ± 2.9 with Ca^2+^, *vs*. 72.4 ± 1.2 without Ca^2+^; n = 3, *P *= 0.1672, *t *= 1.051, df = 4], suggesting that Ca^2+ ^was acting *via *an allosteric mechanism (Fig. 2A,B). This was more evident on a double-reciprocal plot of the data: the slope (*e.g*. K_m_/V_max_) was changed [0.0441 ± 0.0009 (without Ca^2+^: r^2 ^= 0.9973) vs. 0.0328 ± 0.0007 (with Ca^2+^: r^2 ^= 0.9967)], whereas the y-intercept (*e.g*. 1/V_max_) was not [3.13 ± 0.21e^-4 ^(without Ca^2+^) vs. 3.20 ± 0.18e^-4 ^(with Ca^2+^)] (Fig. 2C).

### The Ca^2+ ^ionophore A23187 increases MAO-A activity

**Figure 3:**
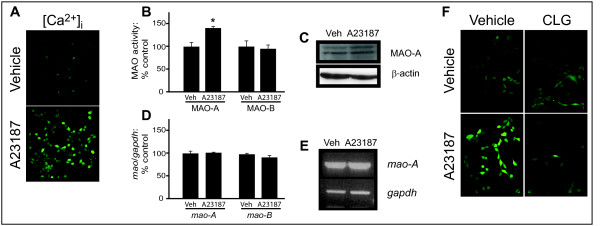
**The Ca^2+ ^ionophore A23187 increases MAO-A activity**. (A) Levels of free intracellular Ca^2+^, [Ca^2+^]_i_, in HT-22 cells treated with A23187 (5 μM: 30 min) were determined using the Ca^2+^-binding Fluo-3 AM fluorescent dye. (B) MAO-A and MAO-B activities were assessed radioenzymatically in A23187-treated cell cultures (*P < 0.05 versus control levels). (C) MAO-A protein expression was determined in SDS-PAGE resolved total cell lysates. Levels of β-actin demonstrate equal protein loading. (D) Densitometry graph representing *mao-A *gene expression (as a ratio to *GAPDH *expression) determined using semi-quantitative RT-PCR amplification of mRNA extracted from A23187-treated cell cultures. (E) Representative RT-PCR amplification fragments. (F) In similarly-treated cells, the production of ROS was assessed using the H_2_O_2_-binding DCF fluorogen. A parallel series of cell cultures were pre-treated with the specific MAO-A inhibitor, clorgyline (CLG; 100 μM, 1 h). Data represent mean ± SD.

HT-22 cells were treated with the Ca^2+ ^ionophore A23187 (5 μM; 30 min) so as to examine the effect of increasing Ca^2+ ^availability on MAO-A activity in living cells. Fluorescence microscopy (Fig. 3A) confirmed a significant increase in free Ca^2+ ^as well as a selective 43% increase in MAO-A activity (control: 55.3 ± 9.4 nmol/h/mg protein versus A23187: 79.1 ± 15.5 nmol/h/mg protein; n = 3, *P *= 0.0424, *t *= 2.270, df = 4) (Fig. 3B). MAO-A protein expression was unchanged compared to control levels (Fig. 3C). The expression of *mao-A *gene (expressed as a ratio to GAPDH) was also not affected by A23187 treatment [control: 100.0% ± 11.2; A23187: 102.0% ± 2.1; n = 3, *P *= 0.7760, *t *= 0.3044, df = 4] (Fig. 3D, E). Neither MAO-B activity [control: 18.92 ± 2.11 nmol/h/mg protein versus A23187: 18.33 ± 1.74 nmol/h/mg protein; n = 3, *P *= 0.5914, *t *= 0.5826, df = 4] nor gene expression [control: 100.0% ± 4.5; with A23187: 92.0% ± 8.2; n = 3, *P *= 0.2127, *t *= 1.481, df = 4] was affected by A23187. An increase in cytoplasmic reactive oxygen species (ROS) was observed, which was partly blocked by pretreatment with the MAO-A inhibitor clorgyline (100 μM; 1 hour) (Fig. 3F).

### Reduction of free intracellular Ca^2+ ^by overexpression of CB-28K reduces MAO-A activity

**Figure 4:**
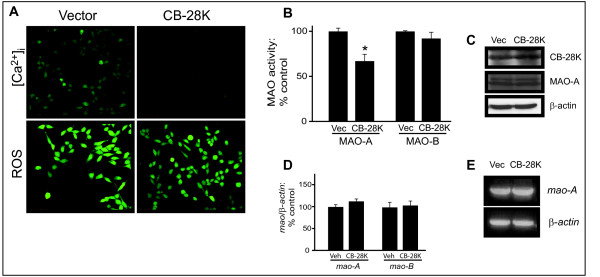
**Overexpression of the Ca^2+^-binding protein CB-28K decreases MAO-A activity**. HT-22 cells were transfected with the pREP plasmid (Vector) or the pREP-CB-28K expression vector (CB-28K). (A) The effect of CB-28K overexpression (24 h) on free intracellular Ca^2+^, [Ca^2+^]_i_, and on ROS production was examined. (*note*, the relative fluorescence intensity used for this set of ROS experiments was increased intentionally so as to avoid a "floor" effect, *i.e*. so that a *decrease *in ROS production could be demonstrated in CB-28K-overexpressing cells). (B) MAO-A and MAO-B activities were assessed radioenzymatically in corresponding cell lysates (*P < 0.05 versus control levels). (C) SDS-PAGE-resolved total cellular proteins were probed for CB-28K overexpression and for the expression of MAO-A and β-actin (used as a loading control). (D) Densitometry graph representing *mao-A *gene expression (relative to β-actin expression) determined by semi-quantitative RT-PCR, represented in (E). Data are presented as mean ± SD.

HT-22 cells were transfected with either the vector control (pREP) or the pREP-CB-28K expression plasmid (Fig. 4). Overexpression of CB-28K (24 h) reduced the level of intracellular free Ca^2+ ^and decreased constitutive ROS levels (Fig. 4A). Overexpressed CB-28K induced a decrease (36%) in basal MAO-A activity [pREP: 69.4 ± 16.4 nmol/h/mg protein versus pREP-CB-28K: 44.4 ± 10.2 nmol/h/mg protein; n = 4, *P *= 0.0440, *t *= 2.246, df = 6] (Fig. 4B). CB-28K overexpression was confirmed by western blot (Fig. 4C) and it did not affect the expression of either *mao-A *gene (expressed as a ratio to β-actin) [with pREP: 100.0% ± 9.8; with pREP-CB-28K: 113.0% ± 9.9; n = 3, *P *= 0.1116, *t *= 1.864, df = 4] or protein (Fig. 4D, E). Neither MAO-B activity [pREP: 8.4 ± 0.47 nmol/h/mg protein versus pREP-CB-28K: 7.0 ± 3.22 nmol/h/mg protein; n = 4, *P *= 0.4226, *t *= 0.8604, df = 6] nor gene expression [with pREP: 100.0% ± 16.1; with pREP-CB-28K: 104.0% ± 17.3; n = 3, *P *= 0.7841, *t *= 0.2930, df = 4] was affected by CB-28K overexpression.

### Immortalized and primary hippocampal toxicity associated with the Alzheimer disease-related peptide, Aβ, relies partly on a MAO-A sensitive mechanism

**Figure 5:**
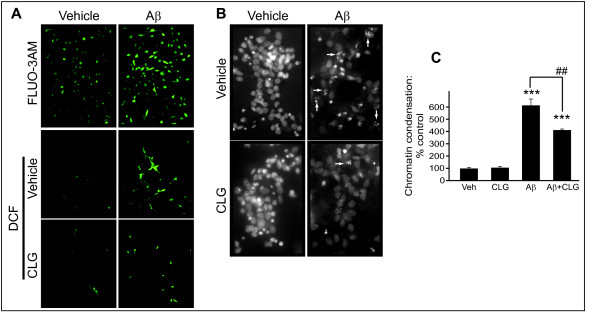
**ROS production induced by the Alzheimer disease-related peptide, Aβ, is diminished by MAO-A inhibition**. HT-22 cells were treated with Aβ(1–40) (30 μM, 48 h). The effect of Aβ(1–40) on (A) free intracellular Ca^2+^, assessed using FLUO-3AM fluorescence, and on the production of ROS, assessed using the H_2_O_2_-binding DCF fluorogen. (B) Hoechst staining of primary hippocampal cell cultures was used to determine the proportion of cells exhibiting chromatin condensation (an indication of apoptotic cell death: arrows) in Aβ(1–42)-treated cultures. In both HT-22 cultures (A) and primary neuronal cultures (B), the effect of Aβ was reversed by pretreatment with the specific MAO-A inhibitor, clorgyline (CLG; 100 μM, 1 h). (C) Number of primary hippocampal cells (expressed as a percentage of control) that exhibit chromatin condensation following treatment with Aβ and/or CLG (groups shown in B). ***: *P *< 0.001 vs. control. ##: *P *< 0.01 between the indicated groups. Data are presented as mean ± SD.

HT-22 cells treated with the AD-related Aβ(1–40) peptide (30 μM; 48 h) had significantly more free Ca^2+ ^(Fig. 5A, top panels) and a corresponding increase in ROS production (Fig. 5A, middle panels) that was decreased by pretreatment with the MAO-A inhibitor clorgyline (100 μM, 1 h) (Fig. 5A, bottom panels). Hoechst staining, which labels all cell nuclei including those that have chromatin condensation (a sign of apoptotic processing), revealed a significant increase in apoptotic primary hippocampal neurons in cultures treated with Aβ(1–42) (10 μM, 24 h) (Fig. 5B) [F_(4,11) _= 69.16, *P *< 0.0001]. *Post-hoc *analysis revealed that the effect of Aβ(1–42) [*P *< 0.001] was greatly attenuated by pre-treatment with the MAO-A inhibitor clorgyline (100 μM; 1 h) [*P *< 0.01] (Fig. 5C).

## Discussion

H_2_O_2 _is a natural by-product of MAO-A-mediated degradation of amines [38]. The normal aging process and various neurodegenerative processes such as Parkinson's disease [39] and Alzheimer's disease [40,41] could well be affected by oxidative stress associated with enhanced MAO-generated levels of H_2_O_2_.

We demonstrate that the selective response of MAO-A to Ca^2+ ^(and its sensitivity to Mg^2+^, a physiologic Ca^2+ ^antagonist [42]) occurs in immortalized HT-22 hippocampal cells and confirms observations using brain extracts from monkey [29], mouse [30], rat [31] and human (DDM: unpublished data). The effect of Ca^2+ ^on MAO-A activity in our cultures appears modest; however, Meyer et al [13] have recently demonstrated that untreated depressed patients have levels of MAO-A density that are only 34% higher than that found in controls. Yet these same authors propose that this modest change could account for most of the change in biogenic amine levels observed in these patients. Obviously, small changes in MAO-A can significantly impact brain function.

While low millimolar levels of Ca^2+ ^are necessary to reveal this effect, the fact that MAO-B activity remains unaffected clearly supports differences in MAO-A and MAO-B regulation. The need for millimolar concentrations also suggests pathological relevance. Interestingly, the mitochondria can modulate cytoplasmic Ca^2+ ^homeostasis by accumulating Ca^2+ ^into the very high micromolar range [43]. Furthermore, elevated Ca^2+ ^concentrations localized to microdomains may also represent unique means of modulating localized mitochondrial membrane and/or matrix function [44], including the activation of dehydrogenases [45]. In neurons and chromaffin cells, mitochondria rapidly and reversibly buffer Ca^2+ ^during cell stimulation to help clear large Ca^2+ ^loads [46-48]. The ensuing overloading of mitochondria with Ca^2+ ^may be involved in several pathological conditions, including ischemia-reperfusion lesions, neurotoxicity and neurodegenerative diseases, where ATP depletion, overproduction of ROS and release of apoptotic factors lead to cell damage [49].

Using the Ca^2+ ^ionophore A23187, we now confirm that increasing free intracellular Ca^2+ ^well above normal levels selectively increases MAO-A activity in living hippocampal HT-22 cells. The associated increase in peroxyradicals is moderately decreased by MAO-A inhibition, supporting a contribution by the enzyme to A23187-associated toxicity. Intracellular Ca^2+ ^levels can also be quenched by specific proteins including calbindin-28K (CB-28K), the predominant Ca^2+^-binding protein in brain [50]. Overexpression of CB-28K in HT-22 cells significantly decreased free Ca^2+ ^levels and significantly decreased MAO-A activity. Again MAO-B activity was spared. The effects of both A23187 and CB-28K occurred independent of any change in the expression level of MAO-A protein (or *mao-A *gene), which, in combination with our demonstration of affinity changes of the MAO-A enzyme in the presence of Ca^2+^, suggests a functional modification of the MAO-A protein itself. These combined data implicate a CB-28K-dependent, Ca^2+^-sensitive component to MAO-A function, which could be exacerbated when CB-28K expression levels are compromised, such as in normal aging or, more importantly, during neurodegenerative processes [16,34,51]. In support of this, an age-related loss of CB-28K immunoreactive basal forebrain cholinergic neurons in cortical regions and in the dentate gyrus (hippocampus) has been shown in several species including dog, mouse and rat [34] and references therein). Furthermore, in AD-related pathology, a substantially greater loss of CB-28K immunoreactivity is observed, with the diminished capacity of the cell to buffer intracellular Ca^2+ ^being considered as a core factor in the pathogenesis of AD [51-54]. This is corroborated by the ability of ectopic CB-28K to protect cell cultures against apoptosis, including that induced by the AD-related peptide β-amyloid [55-58] and by the fact that alterations in Ca^2+ ^signalling can occur early in AD, well before any obvious extracellular Aβ deposition [59]. Furthermore, dihydropyridine Ca^2+ ^channel blockers, which are known to block the selective increase in MAO-A in the senescence-accelerated mouse model [32], may improve learning and performance in animals [60] and may improve age-associated and AD-related memory impairment in humans [61,62]. As CB-28K and MAO-A immunoreactivities are often co-localized and cells immunoreactive for both proteins are reduced during AD [54,63], it is not unreasonable to suppose that a loss of CB-28K could facilitate MAO-A-mediated H_2_O_2 _production in an increasingly toxic Aβ environment, leading to localized cell death. Treatment with Aβ(1–40) increases intracellular levels of Ca^2+ ^[64] [present study], possibly through its effect on the ryanodine receptor [65]. A pathological contribution by MAO-A in AD is further suggested by the accumulation of toxic metabolites of MAO-mediated deamination in AD patients [66] as well as by the ability of MAO-A inhibition to reduce the ROS production associated with treatment of HT-22 cells with the AD-related Aβ(1–40) peptide (present study). Primary hippocampal neuronal cultures are also particularly sensitive to Aβ peptide [67] and Aβ-induced toxicity in these cells is also sensitive to MAO-A inhibition (present study). Experimentation based on *in vivo *assessment of the effect of Ca^2+ ^on MAO-A as well as a closer examination of the relation between Ca^2+^/CB-28K and MAO-A in AD tissues is warranted.

MAO-A is a risk factor in AD and changes in MAO-A activity parallel changes in the production of ROS, *e.g*. H_2_O_2_. Given the neuroprotective role of CB-28K in human pathologies such as AD (and in models of AD), in addition to our demonstration that the toxicity of the AD-related peptide, Aβ, is sensitive to MAO-A inhibition, we suggest that part of the oxidative stress associated with AD may rely on a Ca^2+^/CB-28K-sensitive, MAO-A-mediated mechanism.

## Conclusion

The availability of free intracellular Ca^2+ ^is positively correlated with the activity of MAO-A, a mitochondrial H_2_O_2_-generating enzyme. The influence of Ca^2+ ^on MAO-A function could potentially impact AD-related pathology, which is often associated with altered Ca^2+ ^homeostasis, mitochondrial dysfunction and oxidative stress.

## Competing interests

The author(s) declares that there are no competing interests.

## Authors' contributions

XC carried out the majority of the work. ZW purified and cultured the primary hippocampal cells. GGG participated in the FLUO-3AM microscopy studies. XC, XL and DDM drafted the manuscript. DDM conceived and designed the study, and coordinated data collection and analysis. All authors read, edited and approved the final manuscript.
